# Preliminary examination of the efficacy and safety of a standardized chamomile extract for chronic primary insomnia: A randomized placebo-controlled pilot study

**DOI:** 10.1186/1472-6882-11-78

**Published:** 2011-09-22

**Authors:** Suzanna M Zick, Benjamin D Wright, Ananda Sen, J Todd Arnedt

**Affiliations:** 1 University of Michigan, Department of Family Medicine, 1018 Fuller Street, Ann Arbor, MI 48104-1213, USA; 2 University of Michigan, Department of Neurology and Psychology, 4250 Plymouth Road, Ann Arbor, MI 48109, USA

**Keywords:** Chamomile, *Matricaria*, Insomnia, Herbal medicine, Sleep Quality, Fatigue

## Abstract

**Background:**

Despite being the most commonly used herbal for sleep disorders, chamomile's (*Matricaria recutita*) efficacy and safety for treating chronic primary insomnia is unknown. We examined the preliminary efficacy and safety of chamomile for improving subjective sleep and daytime symptoms in patients with chronic insomnia.

**Methods:**

We performed a randomized, double-blind, placebo-controlled pilot trial in 34 patients aged 18-65 years with DSM-IV primary insomnia for ≥ 6-months. Patients were randomized to 270 mg of chamomile twice daily or placebo for 28-days. The primary outcomes were sleep diary measures. Secondary outcomes included daytime symptoms, safety assessments, and effect size of these measures.

**Results:**

There were no significant differences between groups in changes in sleep diary measures, including total sleep time (TST), sleep efficiency, sleep latency, wake after sleep onset (WASO), sleep quality, and number of awakenings. Chamomile did show modest advantage on daytime functioning, although these did not reach statistical significance. Effect sizes were generally small to moderate (Cohen's *d *≤ 0.20 to < 0.60) with sleep latency, night time awakenings, and Fatigue Severity Scale (FSS), having moderate effect sizes in favor of chamomile. However, TST demonstrated a moderate effect size in favor of placebo. There were no differences in adverse events reported by the chamomile group compared to placebo.

**Conclusion:**

Chamomile could provide modest benefits of daytime functioning and mixed benefits on sleep diary measures relative to placebo in adults with chronic primary insomnia. However, further studies in select insomnia patients would be needed to investigate these conclusions.

**Trial Registration:**

ClinicalTrials.gov Identifier NCT01286324

## Background

Chronic insomnia is a highly prevalent condition, affecting more than 10% of the U.S. population and as many as 33% of adults in primary care settings, where it most commonly presents for initial diagnosis and treatment [1,2]. Persistent insomnia has been linked to reduced quality of life, increased risk for psychiatric and substance use disorders, and exacerbation of accompanying health conditions [3-5]. Estimates also indicate that adults with untreated insomnia incur annual healthcare costs that are more than $1,200 higher than for adults without insomnia [6]. Thus, chronic insomnia exacts a substantial personal and societal burden.

Hypnotic medications, in particular benzodiazepine receptor agonists, and cognitive-behavioral therapy are first-line treatments for chronic insomnia [7]. Despite their demonstrated efficacy in multiple randomized controlled trials, each treatment has significant limitations. For example, while hypnotics are readily available and easy to administer, limited data exist on their efficacy and safety with chronic use, tolerance and dependence may occur with long-term use and many patients prefer non-medication approaches to insomnia. Cognitive-behavioral therapy for insomnia, on the other hand, is effective in both the short- and long-term with few apparent side effects, but accessibility for patients with chronic insomnia is limited.

Studies indicate that a significant proportion of people with insomnia use non-prescription remedies to manage sleep difficulties. In an analysis of the United 2002 National Health Interview Survey Data, 4.5% of the sample of respondents with sleep problems reported using some form of complementary and alternative medicine (CAM) treatment over the preceding 12 months [8]. This percentage represented more than 1.6 million adults with sleep problems in the U.S. population. Morin and colleagues [9] found that 15% of more than 2,000 community-dwelling adults reported using at least one herbal/dietary product for sleep problems in the past year, compared to 11% who reported using a sleep prescription. Yet, rigorous clinical studies assessing the efficacy and safety of CAM therapies for chronic insomnia are largely lacking.

Native to Southern and Eastern Europe and Western Asia, chamomile flowers drunk as teas, delivered in tablets, or applied as oils have been used as medicines for relaxation and for promoting sleep for hundreds of years [10]. German chamomile (*Matricaria recutita*) is also one of the most popular single ingredients in herbal teas and is the most widely used herbal product for sleep [11]. Although its putative mechanism of action is not entirely known, preclinical studies suggest that the flavonoid constituent apigenin produces sedative effects through modulation of y-aminobutryic acid (GABA) receptors [12,13]. Chamomile is generally well tolerated; only a limited number of case reports have documented allergic reactions with its use [14,15]. Only two controlled studies have evaluated the sedative effects of chamomile in humans. Kupfersztain and colleagues [16] found that 12 weeks of an herbal extract for hot flashes that contained chamomile alleviated sleep disturbances and fatigue more than placebo. In another study, adults without sleep complaints who received chamomile jelly had higher peripheral skin temperature, higher ratings of relaxation, and, in men, lower sleep diary ratings of sleep onset latency, nighttime wakefulness, and morning sleepiness more than placebo [17].

In this randomized placebo-controlled pilot study, we evaluated the preliminary efficacy, effect size and safety of four weeks of high-grade chamomile extract in participants with chronic primary insomnia. We hypothesized that subjective measures of sleep quality and the daytime consequences associated with chronic insomnia would improve more with chamomile than placebo. We also expected that chamomile would be equally well tolerated to placebo.

## Methods

Potentially eligible participants were identified through phone calls and emails in response to flyers posted throughout the community and advertisements on Craigslist. The study took place between January 2009 and December 2010. The University of Michigan Medical School Institutional Review Board approved the study protocol and all procedures.

Men and women aged 18 to 65 years of age who met DSM-IV [18] criteria for primary insomnia ≥ 6 months were eligible for screening. We limited the age of the sample to those 18 to 65 because the incidence and causes of insomnia are significantly different in both the younger and older populations, thus requiring unique studies to investigate the effect of chamomile in these populations. We further operationalized insomnia as 7-day sleep diary evidence of a total sleep time (TST) < 6.5 hours and sleep efficiency (SE) < 85% (total sleep time/time in bed*100) on 3 or more nights.

Exclusion criteria included unstable chronic medical conditions, e.g., chronic heart failure, asthma, cancer etc.; current diagnosis of a mood or anxiety disorder, e.g., panic disorder, anxiety, obsessive-compulsive disorder; lifetime history of bipolar or any psychotic disorder; any eating or substance use disorder; evidence of another sleep disorder, such as obstructive sleep apnea or restless legs syndrome; women who were pregnant, lactating, or less than six months post-partum; known allergy or sensitivity to chamomile or members of the ragweed family; and currently taking cyclosporine, warfarin, or any hypnotic medication.

Interested individuals were scheduled for a screening visit. After providing written informed consent, participants underwent a physical exam and medical history, and provided a list of concomitant medications to rule out medical-and substance-related causes of insomnia. Participants also completed a 4-item screener for restless legs syndrome, the Berlin Questionnaire [19] to rule out individuals at high-risk for sleep apnea syndrome, and the Primary Care Evaluation of Mental Disorders, Patient Health Questionnaire (PRIME-MD) a 26-item self-administered questionnaire, which screens for five of the most common psychiatric disorders in primary care: depression, anxiety, alcohol abuse/dependence, somatoform, and eating disorders [20]. Women provided a urine pregnancy test. Physical exam and history were performed by nurse practitioners. However, the study's investigators, a physician and psychiatrist, for study eligibility, evaluated all data obtained from the screening visits. Participants with acceptable screening exams were invited to participate in the study.

Participants who continued to be eligible after sleep diary screening were randomly assigned to receive, *M. recutita *extract, Chamomile High Grade Extract, (MediHerb, [Warwick, Australia]), 270 mg orally twice daily, or a matching placebo. Participants were instructed to take their study capsules between lunch and dinnertime and the second capsule around one hour before bed. Patients were seen at the study clinic 28 days after the baseline visit for their day 28 visit. At the baseline and day 28 visits participants completed their FSS,[21] Beck Depression Inventory (BDI),[22] State Trait Anxiety Inventory,[23] Trait Subscale (STAI-T), Insomnia Severity Index (ISI),[24] and the Pittsburgh Sleep Quality Index (PSQI) [25].

### Objectives and Outcomes

Our primary objective was to compare the effects of four weeks of Chamomile High Grade Extract to placebo on sleep diary measures. Participants completed sleep diaries each morning for one week prior to starting the experimental agent and during the last week of the study (days 21 to 28). The sleep diary was a daily patient log that records bedtime, rise time, sleep onset latency; number and duration of nighttime awakenings, and sleep quality. Total sleep time and sleep efficiency (total sleep time/time in bed*100) were the primary dependent variables derived from the sleep diary, although sleep quality, sleep onset latency, sleep efficiency, number of nighttime awakenings, and wake after sleep onset were also analyzed.

The secondary objectives included: 1) evaluation of common daytime consequences of insomnia, including fatigue and 2) safety and tolerability as assessed by reports at patient visits and weekly phone or email contacts during the 28-day study period. Toxicities were graded based on National Cancer Institute Common Toxicity Criteria version 3.0 for Adverse Events [26].

### Intervention

Eligible participants were randomly assigned to receive a chamomile extract, manufactured by MediHerb (Warwick, Australia), 540 mg (6 tablets daily), or a matching placebo (6 tablets daily). The dose was chosen based on the manufacturer's recommendations and on doses used with chamomile extract that produced a sedative effect in rats [13]. Each capsule contained 90 mg dry extract of chamomile flowering tops [6:1 (v/v) extraction solvent (ethanol 70%/30% water): flowering tops] standardized up to 2.5 mg of (-)-α-bisabolol and ≥ 2.5 mg of apigenin per tablet. The University of Michigan Investigational Drug Service (UM IDS) was responsible for dispensing all study medication. Based on high performance liquid chromatography (HPLC) and gas chromatogram mass spectroscopy (GC-MS) analysis conducted one year after the start of the study, 90 mg of chamomile extract contained 3.9 mg of apigenin and 1.8 mg of (-)-α-bisabolol (Integrated Biomolecule; Tuscon, AZ). Participants were told to take the study medication twice per day with water and to bring all unused tablets to the final (28 day) study visit.

### Randomization, Blinding and Allocation

Eligible participants were randomized equally to either placebo or chamomile groups. The randomization code was computer-generated by the study biostatistician. The randomization list was then given to the research pharmacist who was not associated with the study. The research pharmacist dispensed the study medication, which was in packs provided by the manufacturer, and enclosed it in numbered boxes per the randomization scheme. All study participants as well as all study personnel who assessed outcomes, worked with study data, or administered tests or questionnaires were unaware of the randomization list or treatment assignment. At the final study visits participants were also asked to indicate if they thought they had received placebo or chamomile.

### Statistical Methods and Sample Size

Baseline characteristics are reported, stratified by treatment group, using means and SDs for continuous variables, and counts and percentages for categorical variables. Balance between treatment groups on baseline characteristics was tested using independent samples t-tests for continuous variables and Fisher exact tests for categorical variables.

The effects of study drug versus placebo were evaluated with regression models where the dependent variables were the endpoint of interest at day 28, adjusting for treatment group and baseline value of the variable of interest. For categorical secondary variables, Fisher exact tests were first performed. Between groups effect sizes, reported as Cohen's *d*, were calculated using mean change between baseline and day 28 by group and the pooled standard deviation of the mean change of each treatment group. Analyses were conducted according to the intention-to-treat principle. Data were entered into and analysed with SPSS, Windows version 18 (SPSS, Chicago, ILL). For all analyses, two-sided tests and a significance level of 0.05 were used. No adjustments were made for multiple hypotheses testing as the secondary outcomes were viewed as hypothesis generating.

Since the effect of chamomile on sleep measures had not been studied previously, no such information was available to guide sample size considerations. Thus, our goal was to determine the effect size and variability of chamomile compared to placebo to use for future sample size calculations.

## Results

### Screening, Enrolment, Withdrawals and Adherence

**Figure 1:**
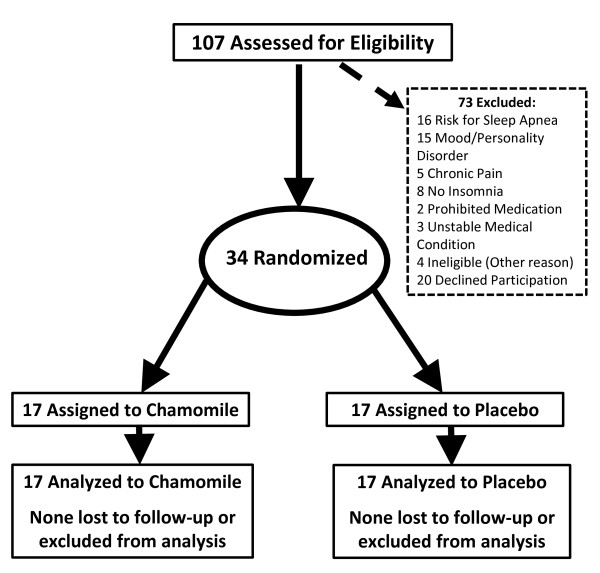
**Participant Flow Through the Study**.

We screened 107 people, of whom 34 met all eligibility criteria and were randomized, 17 to the placebo and 17 to the chamomile group. Figure 1 documents reasons for exclusions. No participants discontinued the intervention. Adherence to study medications was high with 81% of all participants taking greater than 83% of all study medication. Mean intake of tablets was 93% with no significant differences between groups (p = 0.27).

### Sociodemographic Characteristics

In Table 1, we present the sociodemographic and clinical characteristics of participants by treatment group. There were no significant differences between treatment groups for any demographic characteristics.

**Table 1:** Baseline Characteristics of the Randomization Groups

Characteristics	Chamomile *N = 17*	Placebo *N = 17*
Sex, No. (%)		
Men	5 (29)	4 (23)
Women	12 (71)	13 (77)
Race, No. (%)		
White	14 (82)	12 (71)
Age, mean (SD), years	42.2 (13.5)	40.8 (15.3)

### Subjective Measures of Sleep

In Table 2, we present baseline and 28-day values for each subjective sleep measure stratified by group. There were no significant group effects for the primary outcomes, SE% (p = 0.76) or TST (p = 0.11), nor for any of the other sleep diary variables: sleep latency, wake after sleep onset (WASO), number of night time awakenings, and sleep quality. Sleep latency and number of night-time awakenings have moderate effect sizes in favor of chamomile. Chamomile decreased sleep latency by approximately 16 minutes while it was increased by around 1 minute in the placebo group. For number of night time awakenings the placebo group decreased awakenings by 0.3 compared to a 0.8 decrease of night time awakenings in the chamomile group. However, the other sleep outcome with a moderate effect size, TST (0.59 Cohen's *d*) favors the placebo group. The placebo group saw an average increase of 0.8 hours compared to the chamomile group, which increased by 0.2 hours in sleep time between baseline and day 28. Other sleep measures including sleep quality, SE% and WASO demonstrated only small effect sizes (0.06 to 0.23 Cohen's *d*).

**Table 2:** Sleep Diary and Daytime Function Measures by Treatment Group

	Placebo *N = 17* Mean ± SD		Chamomile *N = 17* Mean ± SD			
**Measure**	**Baseline**	**Week 4**	**Baseline**	**Week 4**	**Effect Size^a^**	**P-value^b^**

**Subjective Sleep Measures**						

Sleep Latency (min)	33.9 ± 32.2	32.9 ± 4.3	50.3 ± 53.0	34.2 ± 6.6	0.47	0.41
Wake After Sleep Onset (min)	44.6 ± 46.3	25.9 ± 24.4	49.0 ± 36.6	32.0 ± 27.3	0.06	0.51
Awakenings (#)	1.6 ± 1.1	1.3 ± 0.7	2.2 ± 1.3	1.4 ± 0.6	0.61	0.46
Total Sleep Time (hrs)	5.8 ± 1.0	6.5 ± 1.0	5.9 ± 1.7	6.1 ± 1.2	0.59	0.07
Sleep Quality^c^	3.0 ± 0.7	3.3 ± 0.5	2.7 ± 0.7	3.1 ± 0.5	0.23	0.86
Sleep Efficiency (%)	76.7 ± 12.0	83.3 ± 7.7	71.9 ± 16.7	77.5 ± 12.9	0.09	0.21
ISI Score^d^	13.9 ± 3.6	11.6 ± 4.5	15.1 ± 3.7	11.9 ± 4.7	0.28	0.60
PSQI Total Score^d^	9.5 ± 2.1	7.1 ± 2.7	10.1 ± 2.5	7.5 ± 3.3	0.10	0.88

**Daytime Functioning Measures**						

FSS^d^	30.9 ± 9.1	32.3 ± 10.0	32.1 ± 10.6	27.9 ± 9.3	0.55	0.11
BDI^d^	6.0 ± 6.0	4.8 ± 5.0	3.2 ± 1.6	2.4 ± 2.4	0.31	0.60
STAI						
Trait Subscale^d^	37.5 ± 11.3	40.8 ± 15.5	36.3 ± 10.1	35.5 ± 11.0	0.57	0.45

^a^Effect size is calculated using Cohen's *d*

^b^P-value is derived from a general linear model adjusted for group assignment and baseline value of measure

^c^Sleep quality rated from 1 (very poor) to 5 (very good)

^d ^BDI = Beck Depression Inventory, a score of ≥ 14 is consistent with mild depression, a score of ≥ 20 is consistent with moderate depression and a score ≥ 29 indicates severe depression; ISS = Insomnia Severity Index, 0-7 no clinically significant insomnia, 8-14 is subthreshold insomnia, 15-21 moderate clinical insomnia and ≥ 22 severe clinical insomnia; PSQI = Pittsburgh Sleep Quality Index, scores range from 0-21 with ≥ 6 suggests poor sleep quality; STAI = State Trait Anxiety Inventory, scores range from 20 (no to mild anxiety) to 80 (severe anxiety); FSS = Fatigue Severity Scale, scores range from 9-59 with a score of ≥ 36 indicating significant fatigue and impairment in daytime functioning.

Similarly, there were no significant group effects for changes in the Insomnia Severity Index (ISI) (p = 0.44) or the Pittsburgh Sleep Quality Index (PSQI) total score (p = 0.79). Effect size for each outcome is also presented in Table 1. Both measures favored chamomile compared to placebo, but revealed only small effect sizes in chamomile's favor.

### Assessment of Daytime Consequences of Insomnia

Table 2 presents the results for daytime symptom endpoints. While not statistically significant, we did, however, see a trend in favor of chamomile (p = 0.11) for the FSS. This resulted from a small increase in the fatigue scale in the placebo group (1.4 points or 2.2% increase) and an equally small drop in the fatigue scale in the chamomile group (4.2 points or 6.7% decrease). The difference in the fatigue severity scale represents a moderate effect size of 0.55.

Chamomile had no significant effect on differences in either the BDI (p = 0.60) or the trait subscale of the STAI (p = 0.45). However, the Trait subscale increased by 3.3 points in the placebo group compared to decreasing 0.8 points in the chamomile group representing a moderate effect size of 0.57 favoring chamomile.

### Adverse Events

Adverse events are displayed in Table 3. There were no significant differences in total adverse events (AE) between treatment groups with 10 AE's reported for the placebo group and 6 in the placebo group (p = 0.30). All adverse events were mild (graded as a 1 on the NCI toxicity criteria) and transient. We divided adverse events into categories that most commonly occurred in the trial. There were no statistically significant differences between groups for any of the adverse event categories including headache (p = 0.76), dizziness (p = 1.00), infections (p = 1.00), gastrointestinal symptoms (p = 0.66) or musculoskeletal complaints (p = 0.76).

**Table 3:** Adverse Events By Person (AE's)

	Placebo N(%)	Chamomile N(%)	P-value^a^
Any AE	10 (58.8)	6 (35.5)	0.03
Infections	3 (17.6)	2 (11.8)	1.00
Headaches	1 (5.9)	1 (5.9)	0.76
Dizziness	1 (5.9)	0 (0.0)	1.00
GI Symptoms	4 (23.5)	2 (11.8)	0.66
Musculoskeletal	2 (11.8)	1 (5.9)	0.76

^a^Based on Fisher's Exact Test

### Blinding

We found that study participants were blinded to study assignment. Around 38% of participants believed that they had received chamomile, 32% thought they had received placebo and 29% reported not knowing which treatment they received. There was no significant difference in blinding between treatment groups (p = 0.75).

## Discussion

We found no benefit of a chamomile extract, in the dose and formulation used, on our primary endpoints of subjective sleep efficiency and total sleep time in people with chronic primary insomnia in this preliminary study. We did, however, find a modest benefit of chamomile compared to placebo on other sleep diary measures including sleep latency (0.47 Cohen's *d*), and night time awakenings (0.61 Cohen's *d*), although these did not reach statistical significance. These measures of sleep quality are only slightly smaller than those observed for other drug treatments for chronic insomnia where pooled effect sizes for sleep diary measures (e.g., sleep latency, sleep quality, and wake after sleep onset) ranged from a mean of 0.38 to 0.79 [27].

We observed that those in the chamomile group had a mean decreased sleep latency of a bit more than15 minutes, around 1/3 less night time awakening, and almost a 7% (4.2 point decrease) reduction in the FSS compared to placebo at day 28. These modest changes in sleep latency and night time awakenings are comparable with benzodiazepines, non-benzodiazepines and anti-depressants [27,28]. Where weighted mean differences for decreasing sleep onset latency range from 7.0 to 19.6 minutes depending on the drug studied and how sleep latency was measured [27]. This is despite our study patients, on average, experiencing milder insomnia than those recruited in other drug studies and thus, possibly attenuating our results due to having a less severely affected patient sample.

Despite the popularity of chamomile as a sleep aid [11] there are no published studies examining chamomile for primary insomnia or for any type of chronic sleep disorder. Two other clinical studies have examined the sedative effect of chamomile as a secondary outcome in healthy volunteers and in women with menopausal symptoms [16,17]. Somewhat similar to our results, both of these studies demonstrated that chamomile had positive effects on subjective sleep measures and in the case of menopausal women experiencing hot flashes improvements in fatigue as well. In contrast to our study, which found a mixed effect of chamomile on sleep measures, these studies both reported universally positive effects of chamomile on sleep parameters. However, sleep disturbances in menopausal women experiencing hot flashes may be caused by distinctly different mechanisms than those involved in primary insomnia. Also, it is unclear what improvements in sleep measures indicate in healthy volunteers without sleep disturbances.

Chamomile extract has also been investigated for treatment of stress and generalized anxiety disorders (GAD). Two small studies have evaluated the effect of chamomile essential oil on the autonomic nervous system. Masago and colleagues found that chamomile oil increased comfortable feelings and decreased alpha 1 (8-10 Hz) recordings of the EEG at the parietal and temporal brain regions [29]. Chamomile oil versus placebo oil was also able to significantly shift negative mood images and frequency judgments in a positive direction after being asked to visualize positive or negative phrases [30]. A RCT in people with mild to moderate GAD found that a chamomile extract was significantly better than a placebo extract at decreasing anxiety and showed a positive change in favor of chamomile for changes on the Psychological Well Being and Clinical Global Impression Severity Score [31]. These studies indicate that chamomile may have mild anxiolytic activity, accounting for one mechanism by which it is an effective sleep aid. In our study, we purposely excluded individuals who had diagnoses of depression or anxiety in order to strictly evaluate the effect of chamomile on chronic primary insomnia. As such, we may have excluded the population most likely to experience sleep benefits from chamomile, i.e., those with insomnia that is related to an underlying anxiety disorder.

It is possible that our modest and mixed results were due to an adequate or incorrect; dose, dosing schedule or formulation of chamomile. There has been no dose finding studies with any chamomile product. Consequently, we based our dose on manufacturer recommendations and on doses of apigenin (chamomile's main flavonoid constituent), which produced sedative effects in animal models [12,13,32-34]. Even if a higher dose of apigenin were found to improve sleep, it is not clear that it would be practical to deliver it using chamomile. In our current study our chamomile extract represented 15 gm of chamomile flowers/day. This amount could not feasibly be delivered as a tea and could only be achieved using a concentrated extract. Also, no studies have examined the pharmacokinetics or pharmacodynamics of chamomile, thus severely limiting the data of when and how often chamomile should be administered. Participants in our study took their tablets twice daily. Perhaps more frequent dosing or administration of chamomile through routes other than oral administration could yield better results. While pharmacokinetic studies of chamomile are lacking, other studies indicate that apigenin is orally available and has a reasonable half-life to justify twice daily dosing in humans when delivered in parsley (another rich source of apigenin) [35-37]. Also, it may take longer than four weeks to see larger and more consistent effects of chamomile on sleep and fatigue measures. For instance, the study that found an effect in menopausal women was a 12-week RCT,[16] although if positive effects take longer than one month many insomnia sufferers are likely to abandon the treatment. Moreover, the formulation could have been incorrect. For instance, drinking chamomile as a hot tea, or inhaling it as an essential could produce better results for improving sleep measures. Indeed, the other two studies that found a positive effect of chamomile on sleep measures used different formulations, a chamomile jelly and a tablet, combining chamomile with another herb *Angelica sinensis*.

Chamomile appeared to be well tolerated. There was no difference between placebo and chamomile for all adverse events, for common AE categories including gastrointestinal complaints. No serious adverse events were reported and all non-serious adverse events were mild and transient in nature. Also, our participants were highly adherent taking more than 93% of their tablets on average, indicating that chamomile tablets are both safe and acceptable as a treatment.

Our study had several limitations. First, we were limited by the use of subjective, i.e., pen and pencil, sleep measures. Objective measures of sleep, such as actigraphy or polysomnography, would have been valuable to include and should be used in future studies. Second, we did not assess the characteristics of participants' sleep difficulties, i.e., initial, middle, late or early morning, mixed or non-restorative sleep. These differences in when participants are experiencing sleep issues may be important considering the age-range of individuals included in the study (20-65 years old) and thus, it could be possible that chamomile extract might be more effective for one type or another of sleep difficulties. We were unable to examine these differences in timing due our small sample size with only a few if any participants in any given time category. Also, this was a pilot study and thus we had a small sample size of only 34 participants. However, it was our intention to determine the effect size, direction of effect, and clinical relevance of chamomile on sleep and fatigue outcomes not necessarily statistical significance. Thus, our small number of participants was able to give a good indication of which sleep and fatigue parameters chamomile may benefit and guide future clinical trials in chronic insomnia sufferers. Also, we were able to generate data to determine how many participants would be needed to detect statistically significance differences. For instance, to detect a statistically significant difference in the sleep diary variable with the smallest effect size (0.06 for WASO), a total sample size of 2175 would be required, while only 60 participants (30 per group) would be necessary to detect a significant difference in sleep onset latency (effect size of 0.47).

## Conclusion

In summary, the data from this study points towards the possibility that chamomile extract could provide modest and mixed clinical benefit, at the doses evaluated, to patients with chronic primary insomnia. It is possible, however, that the improvements in the chamomile group are due to events unrelated to treatment, such as natural course of illness and regression toward the mean. Future small studies with chamomile may be warranted in patients with more severe insomnia; those patients whose main insomnia complaints are sleep onset latency or large number of night time awakenings; or patients for whom the side-effects of current insomnia medications are contraindicated or problematic. Also, as chamomile appears beneficial for mild to moderate GAD patients, pilot studies with patients diagnosed with insomnia comorbid with anxiety disorders may be warranted.

## Funding

This research was supported by grants from The University of Michigan Department of Family Medicine Complementary Medicine Seed Grant and the Michigan Institute for Clinical and Health Research (MICHR) NIH UL1RR024986. The funders had no role in the design and conduct of the study; collection, management, analysis, and interpretation of the data; or preparation, review, or approval of the manuscript. No off label drug use was conducted for this study nor did MediHerb offer any financial support or drug for the study. The Chamomile High Grade Extract (MediHerb, Warwick, Australia) used in the study was done so under an investigation IND #101,749 (Suzanna Zick is the IND Sponsor).

This trial is registered as "Chamomile for Chronic Primary Insomnia" in ClinicalTrials.gov ID: NCT01286324

Dr. Zick had full access to all of the data in the study and takes responsibility for the integrity of the data and the accuracy of the data analysis. The authors report no conflicts of interest.

## Competing interests

The authors declare that they have no competing interests.

## Authors' contributions

SZ and JAT drafted the manuscript. AS participated in the design of the study and performed the statistical analysis. SZ and JTA conceived of the study, and participated in its design and coordination. BW participated in its design and coordination. All authors read and approved the final manuscript.

## Pre-publication history

The pre-publication history for this paper can be accessed here:

http://www.biomedcentral.com/1472-6882/11/78/prepub
